# A longitudinal neuroimaging dataset on arithmetic processing in school children

**DOI:** 10.1038/sdata.2019.40

**Published:** 2019-03-05

**Authors:** Macarena Suárez-Pellicioni, Marisa Lytle, Jessica W. Younger, James R. Booth

**Affiliations:** 1 Department of Psychology and Human Development, Vanderbilt University, Nashville, TN, USA; 2 Neurology Department, Neuroscape, University of California San Francisco, San Francisco, CA, USA

**Keywords:** Cognitive neuroscience, Functional magnetic resonance imaging, Human behaviour, Brain imaging

## Abstract

We describe functional and structural data acquired using a 3T scanner in a sample of 132 typically developing children, who were scanned when they were approximately 11 years old (i.e. Time 1). Sixty-three of them were scanned again approximately 2 years later (i.e. Time 2). Children performed four tasks inside the scanner: two arithmetic tasks and two localizer tasks. The arithmetic tasks were a single-digit multiplication and a single-digit subtraction task. The localizer tasks, a written rhyming judgment task and a numerosity judgment task, were used to independently identify verbal and quantity brain areas, respectively. Additionally, we provide data on behavioral performance on the tasks inside the scanner, participants’ scores on standardized tests, including reading and math skill, and a developmental history questionnaire completed by parents. This dataset could be useful to answer questions regarding the neural bases of the development of math in children and its relation to individual differences in skill. The data, entitled “*Brain Correlates of Math Development*”, are freely available from OpenNeuro (https://openneuro.org).

## Background & Summary

Children are expected to successfully develop their mathematic skills to become highly productive adults in a job market that requires a workforce well-trained in science, technology, engineering and mathematics (STEM) disciplines. Efforts should be made in order to understand the reasons why some children fall behind their peers in math, given that they are more likely to have unskilled manual jobs with low pay in the future^1^. Some of these children suffer from dyscalculia, a persistent difficulty in learning math despite normal intelligence that affects about 6% of the childhood population^2^. Although there have been several behavioral studies on math development, little is known about the brain correlates of math skill in children or how the brain supports successful numeric development.

This dataset examines how 8- to 16- year old children process two arithmetic operations that are considered to engage two different brain systems^3,4^: verbal areas for multiplication task solving and magnitude processing areas for subtraction task solving. One hundred and thirty-two children were scanned when they were approximately 11 years old (i.e. mean age; hereinafter, T1) and 63 of them were scanned again approximately 2 years later (hereinafter, T2). At both time points, children solved two arithmetic tasks and two localizer tasks inside the scanner. A single-digit multiplication and single-digit subtraction task constituted the arithmetic tasks and a rhyming judgment and numerosity judgment task constituted the localizer tasks. Children were also administered several standardized tests in order to measure their math fluency, arithmetic ability, attitudes towards mathematics, reading ability, phonological skills, working memory, intelligence and attention deficit hyperactivity disorder. Some of these measures were collected at T1 only, and others at both time points. Additionally, at T1, children’s parents answered a developmental history questionnaire including several questions regarding themselves and about their child’s development, performance in school, and home situation.

There are three mean reasons why this dataset is important and can further contribute to answering relevant scientific questions. First, a significant number of participants in this dataset have longitudinal data. Previous fMRI studies have investigated brain changes associated with math learning by studying how adults learn complex arithmetic problems^5^ or by studying children at different ages^6^. While the cross-sectional approach is able to provide relevant information, the large individual variability in brain structure can fail to detect or falsely suggest changes over time^7^, which constitutes a limitation. There is a consensus in the literature that findings from fully mature brains should not be generalized to children^8^ and that the use of longitudinal designs with children is needed for understanding how the brain supports successful math development^9^. The scarcity of longitudinal fMRI data on math development makes this dataset an important one for answering questions such as whether early brain activation predicts later development or whether brain differences are stable across development.

The second reason why this dataset is an important contribution is the use of localizer tasks. Several previous fMRI studies in math cognition have inferred the engagement of a particular cognitive process using reverse inference. That is, activation in an anatomically defined brain region is attributed to a particular cognitive process using previous literature rather than directly demonstrating the involvement of the region in the cognitive process of interest^10^. By using localizer tasks, we carefully identified brain areas associated with verbal and magnitude processing, allowing stronger conclusions to be drawn. Finally, this dataset provides scores on standardized tests measuring several domain-general cognitive abilities, which would allow future researchers to study brain-behavior correlations. For example, given the wide range of math and reading ability scores in this dataset, questions regarding individual differences in skill can be answered.

This Data Descriptor provides a detailed description of the neuroimaging and behavioral data shared via the OpenNeuro project (https://openneuro.org) and entitled “*Brain Correlates of Math Development*”. This raw data, which was organized in compliance with Brain Imaging Data Structure (BIDS) format, provides the greatest utility and flexibility for reanalysis and new investigations. Part of this dataset has been successfully used in our previous publications. We have used this data to examine developmental, individual and socioeconomic differences in the neural basis of arithmetic^6,11–18^.

## Methods

### Participants

The data set includes data from 132 participants recruited from schools in the Chicago metropolitan area to participate in the study. Participants were primarily recruited through brochures that were sent to many public schools in the Chicago metropolitan area. Other recruitment methods used included print advertisements on public transportation and magazines, electronic advertising on Facebook and Google, and the organization of community events. The brochures were designed to recruit children with a wide range of ability in arithmetic skills, including those who “struggle” with math. A detailed description of the number of participants included in each of the localizer and arithmetic tasks along with the sex distribution is given in Table 1. Participants were screened for eligibility via parent self-report. Exclusionary criteria for this study included: 1) Psychiatric disorders including Attention Deficit Hyperactivity Disorder (ADHD), according to parent report; 2) neurological disease or epilepsy; 3) prematurity less than 36 weeks; 4) birth complications requiring admission into neonatal intensive care unit; 5) head injury requiring emergency medical evaluation; 6) uncorrected visual impairment; 7) significant hearing impairment; 8) non-native English speaker; 9) left-handedness; 10) medication affecting central nervous system processing (e.g. ADHD medication); 11) contraindications for MRI such as having braces. Participant’s unique identification number, sex, age at each time point, data present at each time point, and handedness are shared in a file named *“Participants”.*

**Figure 1:**
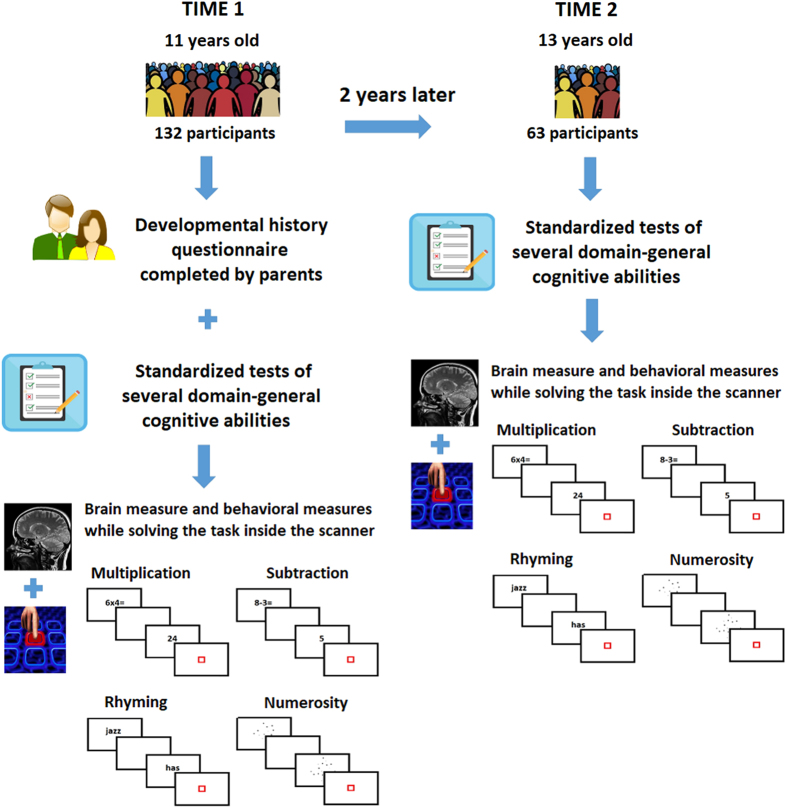
Illustration of the overall study design. Illustration showing the data collected at each time point for the participants having T1 data (n = 132) and for those participants having longitudinal T2 data (n = 63).

Participants were invited back for a longitudinal scan approximately two years after their initial participation at T1. All 132 participants with T1 MRI data were invited for a longitudinal scan, though there was attrition due to moving away from the area, new MRI contraindication (e.g. braces), lack of interest, etc. If participants were no longer eligible for MRI, behavioral data was collected at T2. Of the 132 participants who participated at T1, 63 had longitudinal behavioral and MRI at T2, and an additional 20 longitudinal behavioral data only at T2. More detailed information about the sample is given in Table 2. An overall illustration of the study design is shown in Fig. 1.

Written assent was obtained from the children and consent from their parents or guardians, which included statements referring to the sharing of deidentified data. All experimental procedures were approved by the Institutional Review Board at Northwestern University. These methods are expanded versions of descriptions in our related work^6,11–18^.

### Behavioral assessment

Enrolled participants completed standardized tests in their first visit to the lab, at each time point, in order to measure several domain-general cognitive abilities. Table 4 provides a detailed description of the tests and subtests administered at each time point. Participants first completed the Test of Mathematical Abilities (TOMA-2). All the other tests were completed afterward in counterbalanced order. These data are shared in the *“Phenotype”* subdirectory, as Tab-separated values (i.e. tsv format) together with files describing each of the tests and the developmental history questionnaire (i.e. json). Table 3 provides detailed information about the level of education of the mother and the father, as parents reported in the developmental history questionnaire. Table 5 provides a detailed list of the questions and response options included in the developmental history questionnaire completed by the parents at T1. Table 6 provides a detailed explanation of files location.

### Neuroimaging assessment

#### fMRI acquisition

fMRI data were collected using a Siemens 3T TIM Trio MRI scanner (Siemens Healthcare, Erlangen, Germany) located at the Northwestern University Center for Advanced Magnetic Resonance Imaging (CAMRI) in Chicago, Illinois (USA). The fMRI blood oxygenation level dependent (BOLD) signal was measured with a susceptibility weighted single-shot echo planar imaging (EPI) sequence. A high-resolution T1 weighted 3D structural image (i.e. MPRAGE) was collected for each participant. A 32-channel head coil was used. The scanner parameters for this acquisition were: TR = 2300 ms, TE = 3.36 ms, matrix size = 256 × 256, field of view = 240 mm, slice thickness = 1 mm, number of slices = 160. Full voxel size was 1 × 1 × 1. As for the T2-weighted images, the following parameters were used: TE = 20 ms, flip angle = 80°, matrix size = 128 × 120, field of view = 220 × 206.25 mm, slice thickness = 3 mm (0.48 mm gap), number of slices = 32, TR = 2000 ms, GRAPPA acceleration factor 2. Full voxel size was 1.7 × 1.7 × 3. The first 6 volumes of each run were discarded to allow for T1 equilibration effects.

#### Tasks solved inside the scanner

Participants completed four tasks inside the scanner, two localizer tasks, and two arithmetic tasks. Stimuli were generated using E-prime software (Psychology Software Tools, Pittsburgh, PA) and projected onto a screen that was viewed by the participants through a mirror attached to the head-coil. The timing and order of trial presentation within each run was optimized for estimation efficiency using optseq2 (http://surfer.nmr.mgh.harvard.edu/optseq/). Stimuli were divided into two runs for the numerosity, subtraction, and multiplication tasks. The two runs included different stimuli, containing the same number of items per condition. Trials were displayed to participants in a pseudo-random order within a run, so all participants received trials in the same order within each run. Task and run order were counterbalanced across participants. Behavioral responses were recorded using a two-button keypad below the right hand.

##### Single-digit multiplication task

**Figure 2:**
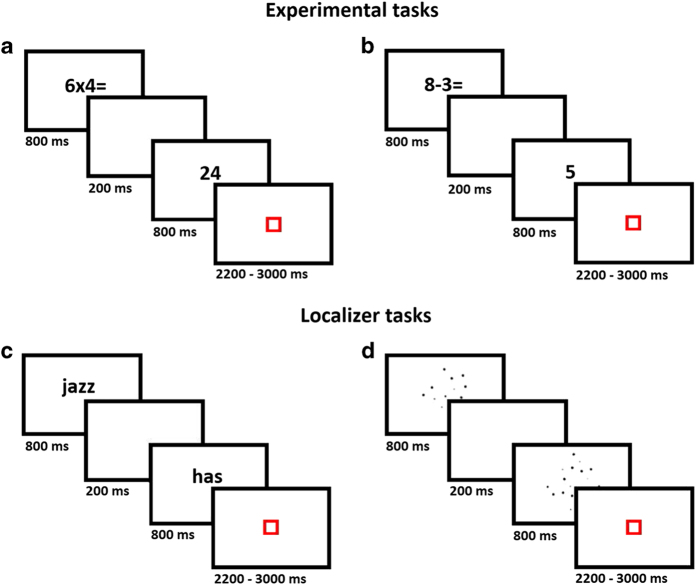
Illustration of tasks stimuli and timing. Illustration of the stimuli and timing for the (**a**) Single-digit multiplication verification task. (**b**) Single-digit subtraction verification task, (**c**) Rhyming judgment task and (**d**) Numerosity judgment task. Parts of this figure have been previously used in our related work ^6,12,13,15,16^.

Participants were presented with 24 single-digit multiplication problems followed by a proposed solution. Half of the problems were classified as “easy” and half as “hard”. Easy problems were characterized as two operands that were less than or equal to 5 (e.g. 2 × 4). Hard problems contained two operands that were larger than 5 (e.g. 9 × 6). Each problem was repeated twice with a correct proposed solution, and once with an incorrect one. Incorrect proposed solutions were the answer to the problem that would be obtained by adding or subtracting 1 to the first operand (e.g. given 9 × 6 = 48, e.g. 9 × 6 = 60). This resulted in 4 different conditions: “Etrue”, easy problems with a correct proposed solution; “Efalse”, easy problems with an incorrect proposed solution; “Htrue”, hard problems with an incorrect proposed solutions and “Hfalse”, hard problems with an incorrect proposed solution. Tie problems (e.g. 5 × 5) and problems involving 0 or 1 were not included. Optseq2 (http://surfer.nmr.mgh.harvard.edu/optseq/) was used for optimization of timing and order of trial presentation. Participants responded with their index finger if the answer to the problem was correct, and with their middle finger if it was incorrect. The multiplication problem (i.e. prime) and proposed solution (i.e. target) were presented on a white background for 800 ms (each) separated by a 200 ms interstimulus interval. The target stimulus was followed by a red fixation square lasting 2200, 2600 or 3000 ms (i.e. 400 ms jitter). Participants could respond as soon as the target was presented, until the beginning of the next trial. In order to control for motor responses, 24 control trials were included, for which a blue square was presented for 800 ms followed by a red fixation square lasting 2200, 2600 or 3000 ms (i.e. 400 ms jitter). Participants’ task was to respond with their index finger when the blue square turned red. The blue and red squares used as control stimuli, common to all the tasks solved inside the scanner, are shared in the subfolder *“Stimuli”.* The total number of trials was 96, which were divided into two separate runs with 48 trials each, entitled *“task-Mult_run-01”* and *“task-Mult_run-02”*. Each run comprised 108 volumes. Each run ended with the presentation of a red square for 22000 ms. Stimuli presentation and timing is shown in Fig. 2a.

##### Single-digit subtraction task

Participants were presented with 24 single-digit subtraction problems followed by a proposed solution. Half of the problems were classified as “easy” and half as “hard”. Easy problems were characterized by a small difference in the first and second operand (i.e. 1, 2 or 3). Hard problems were characterized by a larger difference between the first and second operand (i.e. 4, 5 or 6), and a larger first operand (i.e. 6, 7, 8 or 9). Each problem was repeated twice with a correct proposed solution, and once with an incorrect proposed solution. Incorrect proposed solutions were generated by adding 1 or 2 to the correct answer (e.g. 8 − 2 = 7), or by subtracting 1 from the correct answer (e.g., 8 − 2 = 5). This resulted in 4 different conditions: “Etrue”, easy problems with a correct proposed solution; “Efalse”, easy problems with an incorrect proposed solution; “Htrue”, hard problems with an incorrect proposed solutions and “Hfalse”, hard problems with an incorrect proposed solution. Optseq2 (http://surfer.nmr.mgh.harvard.edu/optseq/) was used for optimization of timing and order of trial presentation. Participants responded with their index finger if the answer to the problem was correct, and with their middle finger if it was incorrect. The subtraction problem (i.e. prime) and the proposed solution (i.e. target) were presented for 800 ms (each) on a white background separated by a 200 ms interstimulus interval. The target stimulus was followed by a red fixation square lasting 2200, 2600 or 3000 ms (i.e. 400 ms jitter). Participants could respond as soon as the target was presented, until the beginning of the next trial. In order to control for motor responses, 24 control trials were included, for which a blue square was presented for 800 ms followed by a red fixation square lasting 2200, 2600 or 3000 ms (i.e. 400 ms jitter). Participants’ task was to respond with their index finger when the blue square turned red. The total number of trials was 96, which were divided into two separate runs with 48 trials each, entitled *“task-Sub_run-01”* and *“task-Sub_run-02”*. Each run comprised 107 volumes. Each run ended with the presentation of a red square for 22000 ms. Stimuli presentation and timing is shown in Fig. 2b.

##### Rhyming judgment task

Participants were presented with two monosyllabic written English words and asked to make a rhyming judgment. Word pairs were categorized into four conditions: 12 pairs were orthographically similar and phonologically similar (O+P+), 10 pairs were orthographically similar and phonologically different (O+P−), 10 pairs were orthographically different and phonologically similar (O−P+), and 14 pairs were orthographically different and phonologically different (O−P−). The number of trials per condition varied slightly due to the use of optseq2 (http://surfer.nmr.mgh.harvard.edu/optseq/) for optimization of timing and order of trial presentation. Participants responded with their index finger if the words rhymed and with their middle finger if they did not rhyme. The first word (i.e. prime) and the second word (i.e. target) were presented for 800 ms (each) on a white background separated by a 200 ms interstimulus interval. The second stimulus was followed by a red fixation square lasting 2200, 2600 or 3000 ms (i.e. 400 ms jitter). Participants could respond as soon as the second word was presented, until the beginning of the next trial. In order to control for motor responses, 25 control trials were included, for which a blue square was presented for 800 ms followed by a red fixation square lasting 2200, 2600 or 3000 ms (i.e. 400 ms jitter). Participants’ task was to respond with their index finger when the blue square turned red. Additionally, 13 perceptual trials were included involving matching judgments of non-alphabetic characters. The characters were presented for 800 ms as increasing, decreasing, or steady in height and subjects were asked to report whether the two presented stimuli matched or did not. Stimuli matched in half of the trials. The stimuli used for this perceptual condition are shared in the subfolder *“Stimuli/task-Rhyming”*. The total number of trials was 84, all presented in one unique run, entitled *“task-Rhyming”.* The run comprised 174 volumes. The run ended with the presentation of a red square for 22000 ms. Stimuli presentation and timing is shown in Fig. 2c.

##### Numerosity judgment task

Participants were presented with two dot arrays. The ratio between the numerosity of the two arrays of dots was varied, such that 24 trials had a ratio of 0.33 (i.e., 12 dots vs. 36 dots; easy condition), 24 trials had a ratio of 0.50 (i.e., 18 dots vs. 36 dots; medium condition), and 24 trials had a ratio of 0.66 (i.e., 24 dots vs. 36 dots; hard condition). A trade-off was used between equating the cumulative surface area and the distribution of single dot sizes (i.e. 6 different dot sizes) in each pair to make sure that participants did not rely on those properties to solve the task. All dot pairs used for this task are shared in the subfolder *“Stimuli/task-Num”.* Optseq2 (http://surfer.nmr.mgh.harvard.edu/optseq/) was used for optimization of timing and order of trial presentation. Participants responded with their index finger if the first array was composed of more dots than the second array, and with their middle finger if the second array was composed of more dots than the first array. The first set of dots (i.e. prime) and the second set of dots (i.e. target) were presented for 800 ms on a white screen separated by a 200 ms interstimulus interval. The target stimulus was followed by a red fixation square lasting 2200, 2600 or 3000 ms (i.e. 400 ms jitter). Participants could respond as soon as the target was presented, until the beginning of the next trial. In order to control for motor responses, 24 control trials were included, for which a blue square was presented for 800 ms followed by a red fixation square lasting 2200, 2600 or 3000 ms (i.e. 400 ms jitter). Participants’ task was to respond with their index finger when the blue square turned red. The total number of trials was 96, which were divided into two separate runs with 48 trials each, entitled *“task-Num_run-01”* and *“task-Num_run-02”.* Each run comprised 107 volumes. Each run ended with the presentation of a red square for 22000 ms. Stimuli presentation and timing is shown in Fig. 2d.

### Data collection: Quality control

Several measures were taken to ensure good data quality. Given that our sample included children, who tend to move inside the scanner more than adults, all subjects participated in a mock fMRI scan approximately one week before the actual fMRI session. This mock session had several objectives, including: 1) carefully explain the task to the child; 2) provide an opportunity for them to practice placement in a fMRI scanner, button box pressing, and the task. For the multiplication and subtraction tasks, twelve problems with a correct proposed solution and twelve problems with an incorrect proposed solution were included in the practice session. For the rhyming and numerosity tasks, twelve trials of each condition were presented in the practice session. Different sets of stimuli were used in the practice and in the scanning sessions; 3) inform participants of the importance of minimizing movement inside the scanner and assess their ability to do so successfully; 4) provide an opportunity for children to familiarize with the fMRI environment in order to reduce anxiety or feelings of discomfort in the actual fMRI session. In the real fMRI session, the examiner reminded the child of the task and button presses via a quick practice session outside the scanner using the same practice stimuli as the mock scanning session, and made sure that the child was comfortable inside the scanner and able to see the screen. Children were encouraged to remain still inside the scanner and were given breaks between the runs in which they could relax and talk to the examiner. Children saw a movie of their choice while the T1-weighted images were collected. All scans were collected by a trained MR technician working with a standardized protocol, who made sure that children were aware and responding to the task and that brain coverage and data quality were good.

## Data Records

This data set is hosted on OpenNeuro (https://openneuro.org), under the accession number ds001486 (Data Citation 1). The files were organized in Brain Imaging Data Structure (BIDS) format^19^ (version 1.1.1; http://bids.neuroimaging.io). The BIDS provides a convention of fMRI data naming and organization in order to facilitate the transfer, storage and sharing of neuroimaging data. The BIDS validation tool provided by OpenNeuro was used to ensure that the dataset was in compliance with the BIDS system.

Participants’ demographic information, including children’s unique identification number, sex, age at each time point and handedness is provided in the *“Participants.tsv”* file and described in the *“Participants.json”* file. The file also includes an indication of the different functional scans available for each participant at each time point. This information was organized into 14 columns containing *“yes”* (data exist) *or “no”* (missing data) for the seven runs at Time 1 (i.e. ses-T1_task-Mult_run-01; ses-T1_task-Mult_run-02; ses-T1_task-Num_run-01; ses-T1_task-Num_run-02; ses-T1_task-Rhyming; ses-T1_task-Sub_run-01: ses-T1_task-Sub_run-02) and at Time 2 (i.e. ses-T2_task-Mult_run-01; ses-T2_task-Mult_run-02; ses-T2_task-Num_run-01; ses-T2_task-Num_run-02; ses-T2_task-Rhyming; ses-T2_task-Sub_run-01: ses-T2_task-Sub_run-02).

The rest of the participants’ data is organized in 4 main folders: 1) *“Phenotype”*: This folder includes participants’ scores on standardized tests at each time point and their parents’ answers to the developmental history questionnaire at T1; 2) *“Stimuli”*: This folder contains the images used as the stimuli used for the Numerosity judgment task, the perceptual condition in the Rhyming judgment task, and those used in the control condition in all the tasks; 3) *“Sub- < ID > ”*: This folder contains participants’ brain and behavioral measures. Inside the folders of each participant with longitudinal data available (i.e. n = 63), there are two subfolders, named *“ses-T1”* and *“ses-T2”*, containing T1 and T2 data, respectively. For those participants with only T1 data available (i.e. n = 69), there is only the folder *“ses-T1”.* Inside each time point subfolder, there are two subfolders named *“anat”* and *“func”*, containing T1-weighted and T2-weighted images, respectively. Finally, inside the *“func”* subfolder, there is a file containing the brain scan (i.e. bold file) and a file containing participant’s performance on the task (i.e. event file). The event file includes both accuracy and response time per trial, as well as onset time of the prime, duration of the event and the presented stimulus. Table 6 provides detailed information on the location of all the shared data at each time point. Table 7 provides information about performance on each task at each time point.

## Technical Validation

Brain imaging data were converted from primary DICOM data to Neuroimaging Informatics Technology Initiative (NIfTI) format using MRIConvert version 2.0. Scanner parameter information was extracted from the DICOM files’ headers and converted to javascript object notation (i.e. json) format.

As part of the process of de-identification of the data, all facial features were removed from T1-weighted anatomical images using mri_deface, an automated program for removing identifying information from structural data^20^. All output images were carefully inspected to ensure that the face removal was complete. This visual check revealed that 81 scans retained some facial information, such as part of the eyes or the tip of the nose. Facial features were removed from these scans using FreeSurfer mri_robust_register to align the raw image to a template space and then performing an inverse registration on a defaced mask^21^. This aligned mask was then multiplied by the raw image to strip all remaining facial features.

**Figure 3:**
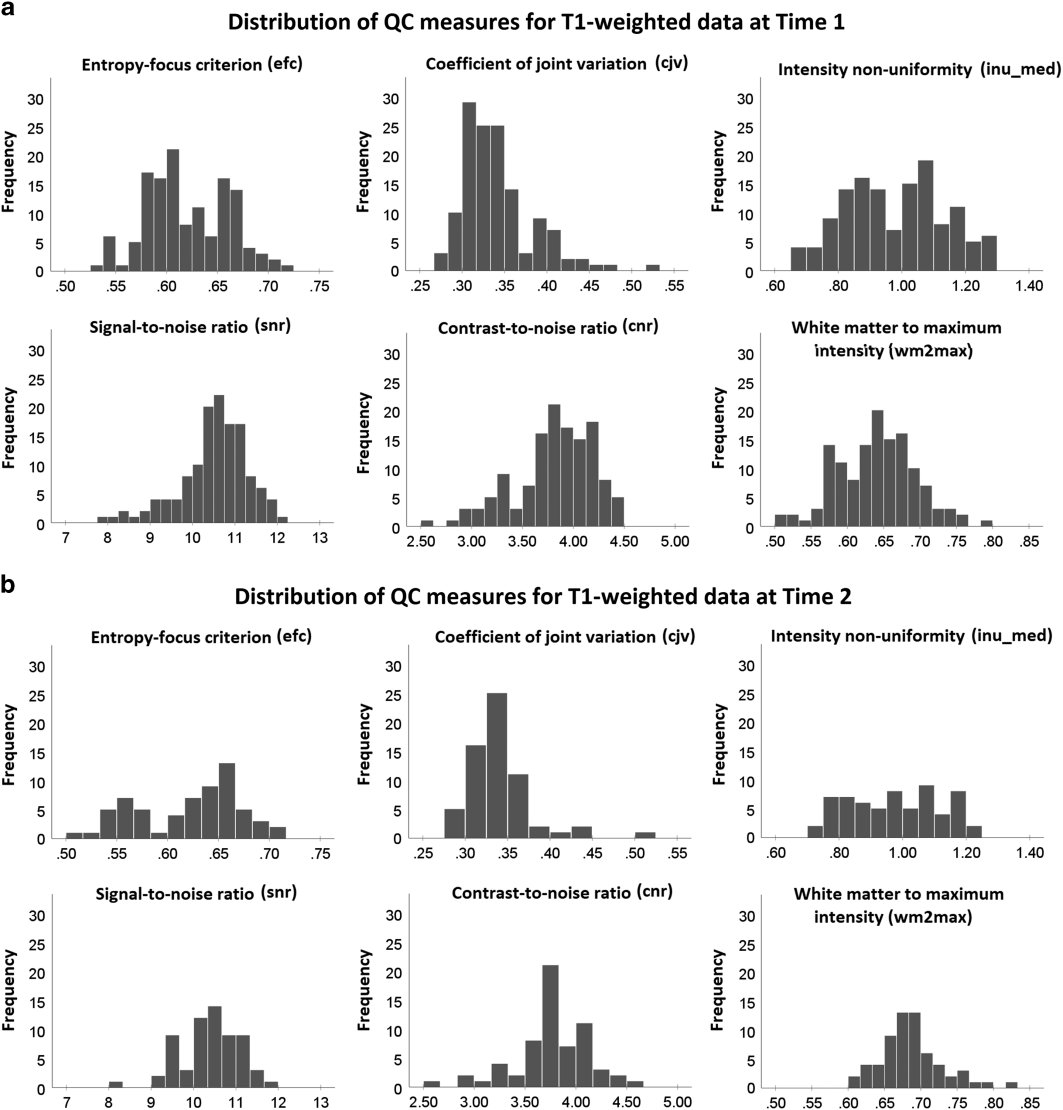
Distribution of T1-weighted data quality metrics. Histograms showing data quality metrics for T1-weighted data for all the runs for all the participants having T1 data (n = 132; **a**) and at T2 for the sample having longitudinal data (n = 63; **b**).

T1-weighted and all runs of T2-weighted data were evaluated with the MRI Quality Control tool (MRIQC; https://github.com/poldracklab/mriqc)^22^. Table 8 provides a description of each of the image quality metrics used to describe both T1-weighted and T2-weighted data. Figure 3 shows histograms providing information on the following image quality metrics for T1-weighted data: Entropy-focus criterion (i.e. efc), Signal-to-noise ratio (i.e. snr), coefficient of joint variation (i.e. cjv), Contrast-to-noise ratio (i.e. cnr), Intensity non-uniformity median (inu_med), White-matter to maximum intensity ratio (i.e. wm2max) both at T1 (a) and at T2 (b). We compared some of these values with the image quality metrics for the Autistic Brain Imaging Data Exchange (ABIDE)’s dataset (http://preprocessed-connectomes-project.org/abide/quality_assessment.html). The Contrast to noise ratio in our data, ranging from 2.50 to 4.50, was lower than the values reported for most of the ABIDE’s data sets, ranging from 5 to 20, but similar to some of its data sets (e.g. CALTECH, OHSU, SBL). As for the entropy-focus criterion, in our data it was slightly larger, ranging from .50 to .70, whereas the majority of ABIDE’s subjects ranged from 0.35 to 0.5. As for the signal-to-noise ratio, ours was slightly slower, ranging from 8 to 12, whereas the majority of ABIDE’s subjects ranged from 5 to 20.

**Figure 4:**
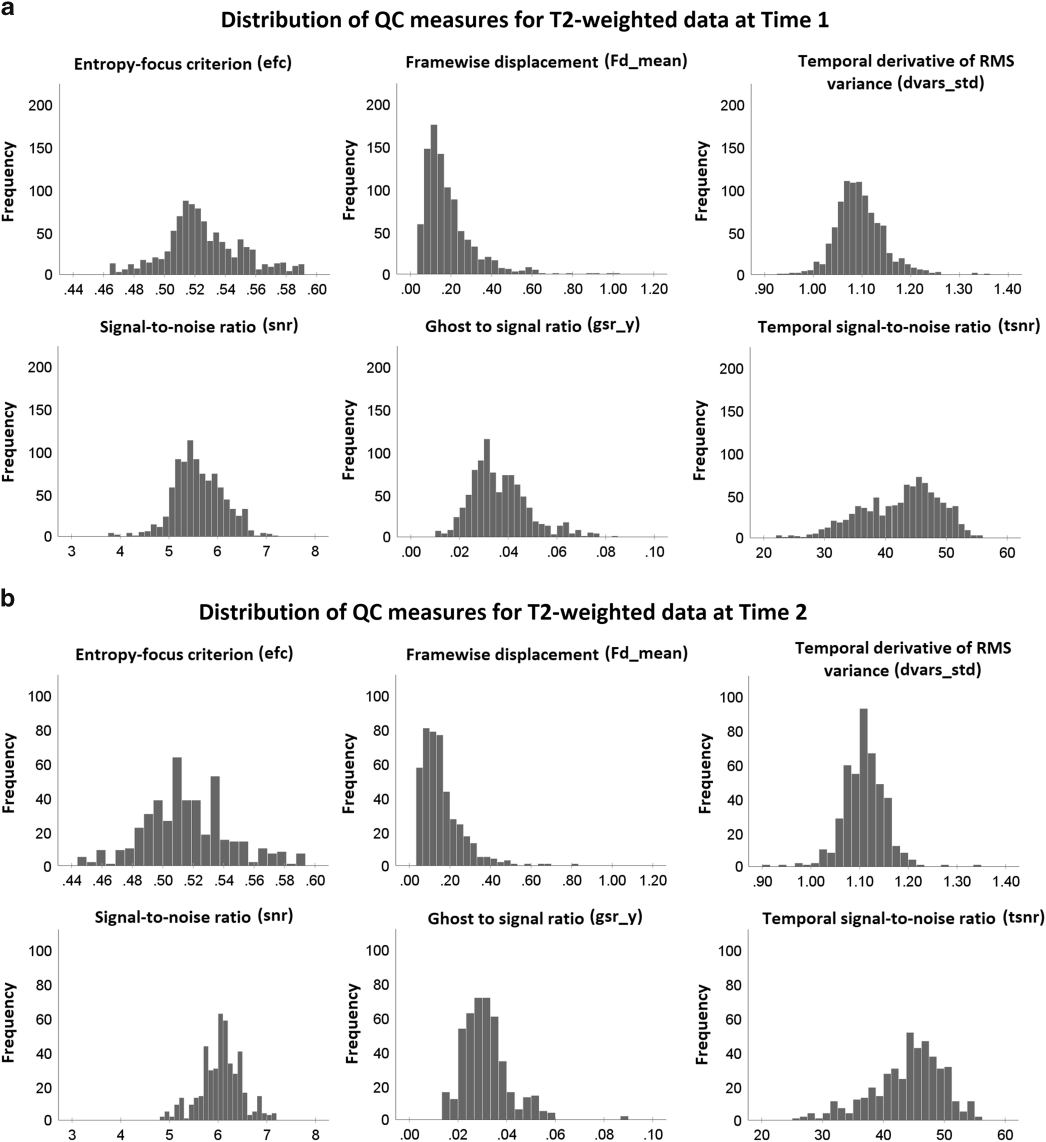
Distribution of T2-weighted data quality metrics. Histograms showing data quality metrics for T2-weighted data for all the runs for all the participants having T1 data (n = 132; **a**) and at T2 for the sample having longitudinal data (n = 63; **b**).

As shown in Fig. 4, we provide histograms describing the following image quality metrics for T2-weighted data: Entropy-focus criterion (i.e. efc), Signal-to-noise ratio (i.e. snr), Temporal derivative of RMS variance over voxels (i.e. dvars_std), Mean framewise displacement (i.e. fd_mean), Ghost-to-signal ratio (i.e. gsr_y) and Median temporal signal-to-noise ratio (i.e. tsnr) both at T1 (a) and at T2 (b). Entropy-focus criterion, Standardized temporal derivative of RMS variance (dvars), Framewise displacement and Ghost to signal ratio were within the range reported for the ABIDE dataset, being 0.4-0.6, 0.8-1.6, 0-0.4 and 0-0.1, respectively.

Additionally, while no participants were excluded for motion, movement was evaluated as an additional gross indicator of data quality. Motion was evaluated with ArtRepair^23^ (http://cibsr.stanford.edu/tools/human-brain-project/artrepair-software/artrepairinstructions.html) to suppress residual fluctuations due to large head motion and to identify volumes with significant artifact and outliers relative to the global mean signal (4% from the global mean). All participants had less than 25% of the total number of volumes replaced in a single run. Only the raw, uncorrected data is available in this dataset. Finally, fMRI event onsets were extracted from the E-prime files using python and are provided in the file named “_events.tsv”, inside each participant’s “func” folder. Each of these event files provides per trial information on the prime and the target presented, the onset time of the prime stimulus, duration of the event, trial type (i.e. condition), accuracy and response time.

## Usage Notes

We encourage other labs to use this dataset for publication under the requirement of citing this article and Data Citation 1 for the source of the data.

## Additional information

**How to cite this article**: Suárez-Pellicioni, M. *et al.* A longitudinal neuroimaging dataset on arithmetic processing in school children. *Sci. Data*. 6:190040 https://doi.org/10.1038/sdata.2019.40 (2019).

**Publisher’s note**: Springer Nature remains neutral with regard to jurisdictional claims in published maps and institutional affiliations.

## Supplementary Material



## Figures and Tables

**Table 1:** Number of participants in each task.

		Number of participants		Number females/males	
Time 1	Time 2	Time 1	Time 2		
Localizers	Rhyming	132	62	70/62	34/28
	Numerosity	132	63	70/62	35/28
Arithmetic	Subtraction	132	63	70/62	35/28
	Multiplication	131	63	70/61	35/28
Number of participants having data for the localizer tasks and the arithmetic tasks at each time point, and sex distribution. Participants having one or more runs of the respective task were included.					

**Table 2:** Sample characteristics.

	T1 sample (n=132)			Longitudinal sample (n=63)					
	Time 1			Time 1			Time 2		
	Mean	SD	Range	Mean	SD	Range	Mean	SD	Range
Age	11.3	1.5	8.4–15.0	11.1	1.5	8.4–14.1	13.4	1.5	10.9–16.5
Reading Fluency	103.1	15.7	59–140	105.6	16.0	59–140	100.6	14.1	55–127
Math Fluency	94.4	15.9	62–143	94.8	16.4	66–143	93.1	16.9	65–138
Intelligence	107.5	15.8	81–144	111.1	16.0	82–144	109.7	17.0	79–144
Mean, standard deviation (SD) and range for age and standardized scores on reading, math fluency and intelligence for participants with Time 1 data (n = 132) and for participants with longitudinal data (n = 63).									
*Note.* Reading Fluency: Word Reading Efficiency standard score on the Test of Word Reading Efficiency (TOWRE); Math fluency: Standardized score on the Math Fluency test from the Woodcock-Johnson III (WJ-III); Intelligence: Full IQ standardized scores on the Wechsler Abbreviated Scale of Intelligence (WASI) (verbal + performance).									

**Table 3:** Level of education of the parents.

Highest degree completed	Mother	Father
No high school	5	7
High school	13	25
Some college	41	32
Bachelor’s degree	35	24
Graduate degree	29	27
No response	9	17
Highest degree of education completed by participants’ mother and father, as reported by parents at T1 in the developmental history questionnaire.		

**Table 4:** Standardized tests at each time point.

Measure	Test	Subtests/Subscales	Time 1	Time 2	Scores
Achievement	Woodcock-Johnson III (WJ-III)	Letter-Word Identification	*		RS & StS
		Word Attack	*		
		Passage Comprehension	*		
		Math Fluency	*	*	
		Spatial Relations	*		
		Basic Reading skills	*		CS
Attention-deficit/hyperactivity disorder	ADHD Rating Scale-IV	Hyperactivity and Impulsivity	*	*	RS & Pe
		Inattention	*	*	
		Total	*	*	
Intelligence	Wechsler Abbreviated Scale of Intelligence (WASI)	Vocabulary	*	*	RS & TS
		Block Design	*	*	
		Similarities	*	*	
		Matrix Reasoning	*	*	
		Verbal IQ	*	*	CS
		Performance IQ	*	*	
		Full IQ	*	*	
Math ability	Comprehensive Math Abilities Test (CMAT)	Addition	*	*	RW & StS
		Subtraction	*	*	
		Multiplication	*	*	
		Division	*	*	
		Basic Calculations	*	*	CS
	KeyMath-3	Measurement	*		RW & ScS
		Foundations of Problem Solving	*		
		Numeration	*	*	
Attitudes	Test of Mathematical Abilities- (TOMA-2)	Attitude Toward Math	*	*	RW & StS
Phonological abilities	Comprehensive Test of Phonological Processing (CTOPP)	Elision	*		RW & StS
		Blending Words	*		
		Rapid Digit Naming	*		
		Rapid Letter Naming	*		
		Phonemic Awareness	*		CS
		Rapid Naming	*		
Reading	Test of Word Reading Efficiency (TOWRE)	Sight Word Efficiency	*	*	RW & StS
		Phonemic Decoding Efficiency	*	*	
Working memory	Automated Working Memory Assessment (AWMA-S)	Digit Recall	*	*	RW & StS
		Listening Recall	*	*	
		Dot Matrix	*	*	
		Spatial Recall	*	*	
List of the standardized tests shared in the data, the cognitive domain measured, the time point at which each measure was collected (T1; T2), the subtests/subscales included and the type of scores available.					
Note. RS: Raw score; ScS: Scale score; StS: Standardized score; CS: Composite score; TS: T-score. Pe: Percentile. T1: Time 1; T2: Time 2.					

**Table 5:** Developmental history questionnaire description.

	Item	Response options
Child’s difficulties	Does your child have speech delays/problems? (i.e.: stutters, difficult to understand)	Yes/No If yes: Please explain
	Was speech/language therapy ever necessary?	Yes/No
	Does your child struggle with reading?	Yes/No. If yes: Please explain
	Has your child ever been tested for a Reading Disability?	Yes/No. If yes, please list diagnosis
	Is or has your child received remediation/tutoring for reading issues?	Yes/No
	Does your child struggle with math?	Yes/No
	Has your child ever been tested for a Math Disability?	Yes/No. If yes, please list diagnosis
	Is or has your child received remediation/tutoring for math issues?	Yes/No
School-related information	What type of school does your child attend?	[Free answer]
	Is your child in a regular classroom?	Yes/No If no, please specify
	Has your child repeated or skipped any grades?	Yes/No If yes, please specify
	Does your child have an Individualized Education Plan or a 504 Plan?	Yes/No
Child’s learning preferences	How does your child prefer to learn?	Listening in class to teacher; Viewing visual information provided in class; Watching demonstrations; Interaction with peers Participating in discussions; Other
Child’s home	Primary language spoken at home:	[Free answer]
	Child lives with:	[Free answer]
Mother’s specific history	Mother’s age:	[Free answer]
	Mother’s occupation:	[Free answer]
	Mother’s highest grade/degree completed:	No High School; High School; Some College; Bachelor’s Degree; Graduate Degree
	Mother: Any history of learning problems:	Yes/No
	Mother: Any history of speech problems:	Yes/No
	Mother: Any history of behavioral problems:	Yes/No
	Mother: Any history of medical problems:	Yes/No
	Mother: Any history of emotional problems:	Yes/No
	Mother: Any history of drug or alcohol abuse:	Yes/No
Father’s specific history	Father’s age:	[Free answer]
	Father’s occupation:	[Free answer]
	Father’s highest grade/degree completed:	No High School; High School; Some College; Bachelor’s Degree; Graduate Degree
	Father: Any history of learning problems:	Yes/No
	Father: Any history of speech problems:	Yes/No
	Father: Any history of behavioral problems:	Yes/No
	Father: Any history of medical problems:	Yes/No
	Father: Any history of emotional problems:	Yes/No
	Father: Any history of drug or alcohol abuse:	Yes/No
	Family: Any history of learning problems:	Yes/No
	Family: Any history of attention deficit disorder:	Yes/No
	Family: Any history of behavioral problems:	Yes/No
	Family: Any history of neurological problems:	Yes/No
Description of the items and the response options included in the developmental history questionnaire completed by the participant’s parents at T1.		

**Table 6:** Summary of data location.

Data type	File name for T1 data	File name for T2 data	Description
Demographics	participants.json	*	Description of the variables included in the participants.tsv file
	participants.tsv	*	Participant’s unique identification number, sex, age, data present at each time point, and handedness
Standardized tests	Phenotype/adhd-rs.json	*	Attention Deficit and Hyperactivity Disorder (ADHD Rating Scale-IV) test and score description
	Phenotype/awma-s.json	*	Automated Working Memory Assessment- Short Form (AWMA-S) test and score description
	Phenotype/cmat.json	*	Comprehensive Math Abilities Test (CMAT) test and score description
	Phenotype/ctopp.json	*	Comprehensive Test of Phonological Processing (CTOPP) test and score description
	Phenotype/developmental_history_questionnaire.json	*	Description of the questions and response options included in the developmental history questionnaire (see Table 5 for a detailed description of the items included)
	Phenotype/keymath-3.json	*	KeyMath-3 test and score description
	Phenotype/toma-2.json	*	Test of Mathematical Abilities- second edition (TOMA-2) test and score description
	Phenotype/towre.json	*	Test of Word Reading Efficiency (TOWRE) test and score description
	Phenotype/wasi.json	*	Wechsler Abbreviated Scale of Intelligence (WASI) test and score description
	Phenotype/wj-III.json	*	Woodcock-Johnson III (WJ-III) test and score description
	Phenotype/ses-T1/adhd-rs.tsv	Phenotype/ses-T2/adhd-rs.tsv	Attention Deficit and Hyperactivity Disorder (ADHD Rating Scale-IV) data at each time point
	Phenotype/ses-T1/awma-s.tsv	Phenotype/ses-T2/awma-s.tsv	Automated Working Memory Assessment Short Form (AWMA-S) data at each time point
	Phenotype/ses-T1/cmat.tsv	Phenotype/ses-T2/cmat.tsv	Comprehensive Math Abilities Test (CMAT) data at each time point
	Phenotype/ses-T1/ctopp.tsv		Comprehensive Test of Phonological Processing (CTOPP) data at T1
	Phenotype/ses-T1/developmental_history_questionnaire. tsv		Developmental history questionnaire data at T1
	Phenotype/ses-T1/keymath-3.tsv	Phenotype/ses-T2/ keymath-3.tsv	KeyMath-3 data at each time point
	Phenotype/ses-T1/toma-2.tsv	Phenotype/ses-T2/toma-2.tsv	Test of Mathematical Abilities- second edition (TOMA-2) data at each time point
	Phenotype/ses-T1/towre.tsv	Phenotype/ses-T2/towre.tsv	Test of Word Reading Efficiency (TOWRE) data at each time point
	Phenotype/ses-T1/wasi.tsv	Phenotype/ses-T2/wasi.tsv	Wechsler Abbreviated Scale of Intelligence (WASI) data at each time point
	Phenotype/ses-T1/wj-III.tsv	Phenotype/ses-T2/wj-III.tsv	Woodcock-Johnson III (WJ-III) data at each time point
Stimuli	Stimuli/task-Num/e.g. dot3_12-1.bmp	*	Stimuli used in the Numerosity localizer task
	Stimuli/task-Rhyming/e.g. perceptual_a.bmp	*	Stimuli used in the perceptual condition of the rhyming localizer task
	Stimuli/e.g. BLUEbox.bmp	*	Stimuli used as control conditions for the localizers and arithmetic tasks
Anatomical scans	T1w.json	*	Scanner parameter information used to acquire structural images.
	Sub- < ID > /ses-T1/anat/sub- < ID > _ses-T1_T1w.nii.gz	Sub- < ID > /ses-T2/anat/sub- < ID > _ses-T2_T2w.nii.gz	Structural images (MPRAGES) at each time point
Functional scans	task-Mult_bold.json	*	Description of the multiplication task solved inside the scanner
	task-Sub_bold.json	*	Description of the subtraction task solved inside the scanner
	task-Num_bold.json	*	Description of the numerosity task solved inside the scanner
	task-Rhyming_bold.json	*	Description of the rhyming task solved inside the scanner
	Sub- < ID > /ses-T1/func/sub- < ID > _ses-T1_task- < name > _run- < num > _bold.nii.gz	Sub- < ID > /ses-T2/func/sub- < ID > _ses-T2_task- < name > _run- < num > _bold.nii.gz	Nifti raw data at each time point
Behavioral responses	Sub- < ID > /ses-T1/func/sub- < ID > _ses-T1_task- < name > _run- < num > _events.tsv	Sub- < ID > /ses-T2/func/sub- < ID > _ses-T2_task- < name > _run- < num > _events.tsv	Stimuli, onset time of the prime, duration, trial type, accuracy and response time per each trial of each run at each time point
Notes	README	*	Explanation of known issues with the data
Type of data, location for each time point and description of the information included in each location.			
Note. Json: javascript object notation; Tsv: Tab-separated value; bmp: bitmap image; .nii.gz: nifty gzip-compressed. (*) Common to both time points.			

**Table 7:** Performance on the tasks solved inside the scanner.

	T1 sample (n=132)		Longitudinal sample (n=63)			
	Time 1		Time 1		Time 2	
	Accuracy	Response times	Accuracy	Response times	Accuracy	Response times
Multiplication	78.2 (41.3)	1080 (610)	79.5 (12.9)	1125 (349)	82.1 (38.3)	1036 (528)
Subtraction	76.5 (42.4)	1222 (593)	75.9 (18.0)	1306 (366)	88.2 (32.3)	1034 (520)
Rhyming	75.3 (43.1)	1228 (439)	77.5 (14.1)	1261 (255)	84.5 (36.2)	1083 (379)
Numerosity	84.7 (36.0)	1020 (439)	85.3 (10.9)	1040 (263)	91.8 (27.4)	873 (400)
Percentage of accuracy and mean of response times for correctly solved trials (standard deviation in parenthesis) for all the experimental conditions at each time point.						

**Table 8:** Quality metrics description.

Image Type	Metric	Description
Both	Entropy-focus criterion (efc)	Indication of blurring and ghosting caused by head motion. Lower values are better^24^.
	Signal-to-noise ratio (snr)	A measure of quality of signal sensitive to the MRI system and parameters. Higher values are better^25^.
T1-weighted	Coefficient of joint variation (cjv)	A measure of the performance of INU correction. Higher values indicate greater amounts of head motion and large INU artifacts. Lower values are better^26^.
	Contrast-to-noise ratio (cnr)	A measure of the contrast between white matter and gray matter. Higher values are better^25^.
	Intensity non-uniformity median (inu_med)	Location and spread of the bias field from INU correction. Values closer to 1.0 are better^27^.
	White-matter to maximum intensity ratio (wm2max)	Median intensity of white matter over the 95^th^ percentile of full intensity distribution. Values between 0.6 and 0.8 are best^22^.
T2-weighted	Mean framewise displacement (fd_mean)	Measure of head movement across data acquisition calculated by realignment estimates. Lower values are better^28^.
	Ghost-to-signal ratio (gsr_y)	The intensity of Nyquist ghost signal in the y-direction due to suboptimal EPI sequence calibrations. Lower values are better^29^.
	Normalized temporal derivative of RMS variance (dvars_std)	Standardized measure of the intensity change across volumes. Lower values are better^30^.
	Median temporal signal-to-noise ratio (tsnr)	Median value of signal across time over temporal standard deviation. Higher values are better^22^.
Image quality metric used to assess T1-weighted and T2-weighted data quality and their description, including information on how to interpret each of the values.		
